# 

**Published:** 1989-11

**Authors:** 


Copyright © Clinical Press Ltd 1989.

**Contents**

89 **Editorial**
90 **Impressions**
A. John Webb
96 **Obituary—Peter Campbell Clifford**
97 **Carey Franklin Coombs 1879–1932**
Clive F. M. Weston
104 **Inguinal Hernia Waiting Lists; Medical and Financial Implications**
A. H. Davies, J. Mountfield, C. P. Armstrong
105 **A case of Self-Inflicted Penile Ulceration**
D. S. Allen
107 **From Our Correspondents**
109 **Leather**
J. M. A. Smith
112 **South West Orthopaedic Club**
Meeting at Newport, November, 1988
115 **Index for 1989**

